# Hydroxycarboxylic Acid Receptor 1 and Neuroprotection in a Mouse Model of Cerebral Ischemia-Reperfusion

**DOI:** 10.3389/fphys.2021.689239

**Published:** 2021-05-21

**Authors:** Lara Buscemi, Camille Blochet, Pierre J. Magistretti, Lorenz Hirt

**Affiliations:** ^1^Stroke Laboratory, Neurology Service, Department of Clinical Neurosciences, Lausanne University Hospital, University of Lausanne, Lausanne, Switzerland; ^2^Department of Fundamental Neurosciences, University of Lausanne, Lausanne, Switzerland; ^3^Division of Biological and Environmental Sciences and Engineering, King Abdullah University of Science and Technology, Thuwal, Saudi Arabia

**Keywords:** metabolism, middle cerebral artery occlusion, ischemia, neuroprotection, lactate, hydroxycarboxylic acid receptor 1

## Abstract

Lactate is an intriguing molecule with emerging physiological roles in the brain. It has beneficial effects in animal models of acute brain injuries and traumatic brain injury or subarachnoid hemorrhage patients. However, the mechanism by which lactate provides protection is unclear. While there is evidence of a metabolic effect of lactate providing energy to deprived neurons, it can also activate the hydroxycarboxylic acid receptor 1 (HCAR1), a Gi-coupled protein receptor that modulates neuronal firing rates. After cerebral hypoxia-ischemia, endogenously produced brain lactate is largely increased, and the exogenous administration of more lactate can decrease lesion size and ameliorate the neurological outcome. To test whether HCAR1 plays a role in lactate-induced neuroprotection, we injected the agonists 3-chloro-5-hydroxybenzoic acid and 3,5-dihydroxybenzoic acid into mice subjected to 30-min middle cerebral artery occlusion. The *in vivo* administration of HCAR1 agonists at reperfusion did not appear to exert any relevant protective effect as seen with lactate administration. Our results suggest that the protective effects of lactate after hypoxia-ischemia come rather from the metabolic effects of lactate than its signaling through HCAR1.

## Introduction

Lactate, a presumed trivial metabolite of glycolysis, has gained a lot of interest in neuroscience in recent years. On the one hand, lactate is a preferred energy substrate of neurons and can be provided to neurons by astrocytes (Pellerin and Magistretti, 1994); on the other hand, lactate acts as a volume transmitter and mediator of metabolic information in the neurovascular unit [For review: (Bergersen and Gjedde, 2012)]. Lactate plays a critical physiological role in long-term memory formation and plasticity (Suzuki et al., 2011; Yang et al., 2014) and has been shown to have beneficial effects in animal models of acute brain injuries, including ischemic stroke (Rice et al., 2002; Berthet et al., 2009, 2012; Castillo et al., 2015; Zhou et al., 2018; Buscemi et al., 2020), and in patients suffering from traumatic brain injury or subarachnoid hemorrhage (Bouzat et al., 2014; Carteron et al., 2018). However, the mechanism by which lactate provides neuroprotection is not clear.

While there is evidence that lactate has a beneficial metabolic effect providing energy to deprived neurons (Schurr et al., 1997, 1999; Roumes et al., 2020), it can also activate the hydroxycarboxylic acid receptor 1 (HCAR1), a Gi-coupled protein receptor (Cai et al., 2008; Ahmed et al., 2009) that modulates neuronal firing rates (Bozzo et al., 2013; Herrera-Lopez and Galvan, 2018; de Castro Abrantes et al., 2019). After cerebral hypoxia-ischemia, endogenously produced brain lactate is largely increased (Harada et al., 1992; Lei et al., 2009; Alf et al., 2012; Hyacinthe et al., 2020), and the exogenous administration of more lactate, systemically or directly into the brain, can decrease the lesion size and ameliorate the neurological outcome (Berthet et al., 2009, 2012; Castillo et al., 2015; Buscemi et al., 2020; Roumes et al., 2020). As lactate could be used as a metabolic substrate and/or trigger a signaling response after binding to its receptor, a dual mechanism of action was envisaged for lactate neuroprotection. Indeed, there is *in vivo* evidence that exogenous lactate administered after ischemia can quickly reach the brain and is converted into pyruvate and further oxidized (Hyacinthe et al., 2020; Roumes et al., 2020). Also, we have *in vivo* and *in vitro* evidence that D-lactate exerts neuroprotection, and *in vitro* evidence that the activation of the Gi-coupled lactate receptor without any direct effect on metabolism can have a neuroprotective effect after oxygen and glucose deprivation (OGD; Castillo et al., 2015).

While lactate receptor agonists have been tested using several *in vitro* paradigms (Dvorak et al., 2012; Liu et al., 2012; Bozzo et al., 2013; Lauritzen et al., 2014; Castillo et al., 2015; Herrera-Lopez and Galvan, 2018; Vardjan et al., 2018; de Castro Abrantes et al., 2019; Vohra et al., 2019), their effects have been only recently tested in the central nervous system with unclear results (Lev-Vachnish et al., 2019; Scavuzzo et al., 2020). To further investigate in this direction and shed some light on a presumable dual protective effect of lactate, we tested if HCAR1 stimulation could provide neuroprotection when administered *in vivo* in a mouse model of transient hypoxia-ischemia. With this aim, we evaluated the effect on the ischemic lesion size and the neurological outcome of two different compounds, the synthetic 3-chloro-5-hydroxybenzoic acid (CHBA) and 3,5-dihydroxybenzoic acid (DHBA), a metabolic product of the β-oxidation of alkylresorcinols found in cereals such as rye and wheat (Ross et al., 2004), both of them considered HCAR1 full agonists.

## Materials and Methods

### Transient Middle Cerebral Artery Occlusion Model

For our *in vivo* experiments, we used a well-established mouse transient middle cerebral artery occlusion (MCAO) model of ischemic stroke with reperfusion (Longa et al., 1989) on 8–10 weeks male C57BL/6 J mice (Charles River, France). The veterinary authorities of Canton Vaud according to the Federal guidelines of the Swiss Veterinary Office approved the experimental and surgical procedures. Mice had *ad libitum* access to food and water and were housed under standard conditions, except for postsurgical recovery, when they were kept overnight in their home cages inside a temperature-controlled incubator at 28°C. Ischemia was induced by inserting a silicone-coated suture (Doccol) through the left common carotid artery into the internal carotid artery and advancing it into the arterial circle to occlude the origin of the middle cerebral artery. After 30 min, the coated suture was withdrawn and the cerebral blood flow (CBF) restored. The whole procedure was done under laser Doppler CBF control, maintaining the rectal temperature at 37 ± 0.5°C. The surgery was considered successful if the CBF during occlusion reached below 20% of the initial value and reperfusion reached at least 50% of the initial value within 10 min of filament removal. Mice received subcutaneous injections of buprenorphine (0.025 mg/kg) pre- and post-surgery for analgesia.

### Lesion Volume Measurement

**Figure 1:**
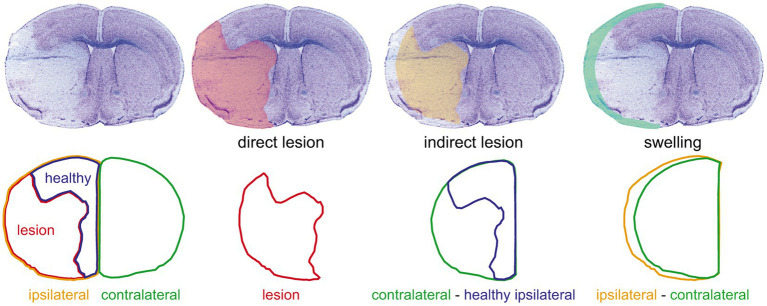
Evaluation of the ischemic damage. The upper row shows (from left to right) a representative image of a cresyl violet-stained coronal section of a 48 h post-middle cerebral artery occlusion mouse brain and the overlays on the same section of the corresponding direct lesion (red shading), indirect lesion (yellow shading), and swelling (light green shading). The lower row shows the outlines of the different areas considered [ipsilateral hemisphere area (yellow outline), lesion area (red outline), healthy ipsilateral area (blue outline), and contralateral hemisphere area (green outline)] and the operations used to measure the damage illustrated in the corresponding upper row. This procedure was done on a set of 12 serial sections per brain to estimate the lesion and swelling volumes (see “Materials and Methods” section).

Lesion volumes were measured from cresyl violet-stained serial coronal (20-μm thick and 720-μm apart) cryostat sections of liquid nitrogen vapor-frozen tissue obtained 48 h after MCAO surgery (Buscemi et al., 2020; Figure 1). Images were taken with a Nikon SMZ25 stereomicroscope and were measured while blinded to the treatment using ImageJ. The direct infarct size was calculated as the sum of the infarcted areas on each section multiplied by the spacing distance between sections. The indirect infarct size was calculated as the subtraction of the healthy ipsilateral volume from the contralateral volume. The percentage of cerebral hemisphere swelling was calculated as the difference between ipsilateral and contralateral volumes divided by the total brain volume.

### Functional Outcome Assessment

We assessed the functional outcome of mice by neurological score evaluation, Rotarod test, and/or wire-hanging test (Blochet et al., 2020; Buscemi et al., 2020). We performed the assessment and analysis of the behavioral outcomes blinded to the treatment.

We evaluated the neurological deficit on all mice immediately after surgery and at 24 h and 48 h after MCAO and graded for severity using the following scale: 0 for no observable deficit, 1 for failure to extend the forepaw, 1.5 for intermittent circling, 2 for persistent circling, and 3 for loss of circling or righting reflex.

A set of mice were subjected to the Rotarod test, where they were placed on an accelerating (4 to 40 rpm) rotating cylinder (Ugo Basile) and left to run for a maximum of 900 s. We trained mice for three consecutive days before surgery and tested them 24 h and 48 h after MCAO. The longest latency to fall from the rod over three trials was scored for each time point.

We subjected all mice to the wire-hanging test, where they were suspended on a single wire stretched between two posts above a soft ground and allowed to escape toward the posts. We trained mice 1 day before intervention and scored for escaping and/or falling events at 24 h and 48 h after MCAO. The test lasted for a maximum of 180 s on the wire or a maximum of 10 falls. We evaluated the overall performance as (from better to worse) only escape, neither escape nor fall, escape or fall, and only fall. We also evaluated the number of falls, the number of escapes, and the maximal latency time to fall from the wire. We used The Observer software (Noldus) for data extraction.

### Lactate Receptor Agonist Treatments

Mice with successful reperfusion were injected with either the HCAR1 agonists (CHBA or DHBA) or with vehicle (PBS). Only a single intravenous (IV) or intracerebroventricular (ICV) injection of the agonist or vehicle was given, 15–20 min after filament removal. We administered the treatments in a randomized fashion by an investigator blinded to the treatment. The doses were chosen as explained in the results.

#### IV Injections

For the pilot dose-response of CHBA IV injection, the agonist was used at 0.1 μmol/g or 0.025 μmol/g. We used 25 mice for this experiment; two mice were discarded due to insufficient reperfusion and six mice due to unsuccessful IV injection. An experiment with a larger number of animals per group was done with an IV injection of 0.1 μmol/g CHBA or vehicle. For this experiment, 30 mice were used; six mice were discarded due to insufficient reperfusion and two mice due to unsuccessful IV injection.

#### ICV Injections

For the ICV administration of the CHBA experiment, 2 μl of 1 mmol/L CHBA (total dose 2 nmol per mouse) or PBS were delivered into the left ventricle [0.9 mm laterally, 0.1 mm posteriorly, and 3.1 mm deep from bregma (Hirt et al., 2004)]. We used 16 mice for this experiment; one mouse was discarded due to insufficient reperfusion and one mouse injected with PBS had to be sacrificed before the end of the experiment. For the ICV administration of DHBA experiment, 2 μl of 100 mmol/L DHBA (total dose 0.2 μmol per mouse) or PBS were delivered into the left ventricle (see above). We used 18 mice for this experiment; one mouse was discarded due to insufficient reperfusion, one mouse injected with PBS had to be sacrificed before the end of the experiment, and two more mice were discarded due to failed injection. One of the animals had a swollen hind limb (unrelated to the MCAO) and was not considered fit for the behavioral test.

### Statistical Analysis

We compared continuous variables following Gaussian distributions with one-way ANOVA with Tukey’s *post-hoc* test (multiple groups) or with unpaired two-tailed Student’s *t*-test (two groups). We compared non-Gaussian distributions, with the Kruskal–Wallis test. For analysis across time, we used two-way ANOVA. Statistical tests were run on GraphPad Prism 6.0. On the box-and-whisker plots, the line shows the median and the whiskers correspond to the maximum and minimum values. On the floating bar plots, the line shows the mean value. We considered significance as *p* < 0.05.

## Results

**Figure 2:**
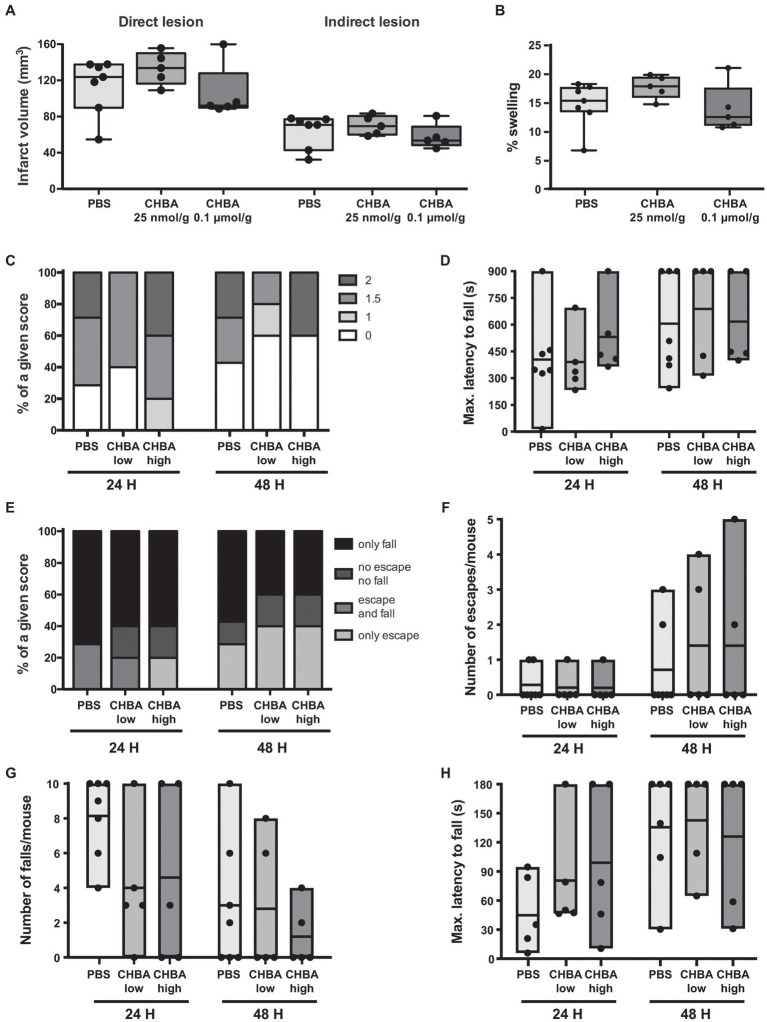
Effect of the intravenous (IV) administration of two different doses of the lactate receptor agonist 3-chloro-5-hydroxybenzoic acid (CHBA) after 30 min MCAO. **(A)** Direct (left) and indirect (right) lesion volumes and **(B)** percentage of swelling from brains of mice that received an IV injection of vehicle (PBS, *n* = 7), a low dose (25 nmol/g, *n* = 5) or a high dose (0.1 μmol/g, *n* = 5) of CHBA at reperfusion. Measurements were done on cresyl violet-stained serial coronal sections of frozen brains from mice sacrificed 48 h after MCAO induction. To evaluate the functional outcome following IV treatment with PBS or CHBA, mice were scored for **(C)** neurological deficit (neuroscores: 0, no deficit; 1, failure to extend right forepaw; 1.5, intermittent circling; 2, circling; and 3, loss of circling or righting reflex), **(D)** performance on Rotarod, **(E)** overall performance on wire-hanging test, **(F)** number of falls from the wire, **(G)** number of times mice escaped from the wire, and **(H)** maximal latency time to fall from the wire. All the behavioral measurements were recorded at 24 h and 48 h after MCAO. Filled dots represent individual animals.

To better understand the role of the lactate receptor HCAR1 in lactate-induced neuroprotection, we started by testing the potent agonist CHBA. The apparent EC_50_ of CHBA acting on HCAR1 calculated from *in vitro* data is 0.02 mM (Dvorak et al., 2012; de Castro Abrantes et al., 2019), 100–250 times lower than the effective lactate concentrations used *in vitro* (Cai et al., 2008; Liu et al., 2009). To the best of our knowledge, there is no published information on the pharmacokinetics (bioavailability, permeability, or transport) of any of the HCAR1 agonists. We chose therefore two CHBA doses to test based on the only report showing *in vivo* effects of this agonist after intravenous administration in mice, of 0.15 μmol/g (Wallenius et al., 2017). In an exploratory experiment, we injected intravenously 0.1 μmol/g CHBA, 0.025 μmol/g CHBA or vehicle to mice subjected to 30-min MCAO, shortly after reperfusion (Figure 2).

**Figure 3:**
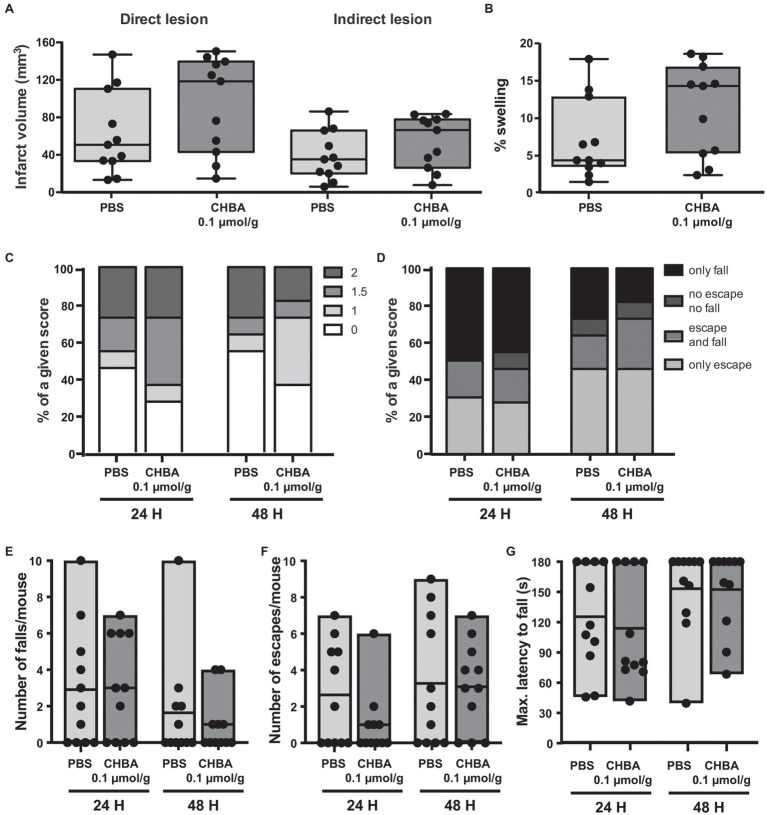
Effect of the intravenous (IV) administration of 0.1 μmol/g of the lactate receptor agonist CHBA after 30 min MCAO. **(A)** Direct (left) and indirect (right) lesion volumes and **(B)** percentage of swelling from brains of mice that received an IV injection of vehicle (PBS, *n* = 11) or 0.1 μmol/g of CHBA (*n* = 11) at reperfusion. Measurements were done on cresyl violet-stained serial coronal sections of frozen brains from mice sacrificed 48 h after MCAO induction. To evaluate the functional outcome following IV treatment with PBS or CHBA, mice were scored for **(C)** neurological deficit (neuroscores: 0, no deficit; 1, failure to extend right forepaw; 1.5, intermittent circling; 2, circling; and 3, loss of circling or righting reflex), **(D)** overall performance on wire-hanging test, **(E)** number of falls from the wire, **(F)** number of times mice escaped from the wire, and **(G)** maximal latency time to fall from the wire. All the behavioral measurements were recorded at 24 h and 48 h after MCAO. Filled dots represent individual animals.

We did not observe any significant change in lesion size or behavioral outcome with any of the two tested doses. However, despite the low number of animals used, there appeared to be a trend for a beneficial effect on lesion size and early outcome at the higher dose of CHBA, whereas the lower dose was suggestive of larger lesion sizes. Then, we performed a second experiment using 0.1 μmol/g CHBA on a larger number of animals (Figure 3). With this larger sample size, we did not observe any significant effect of the agonist treatment on lesion size or neurological outcome.

**Figure 4:**
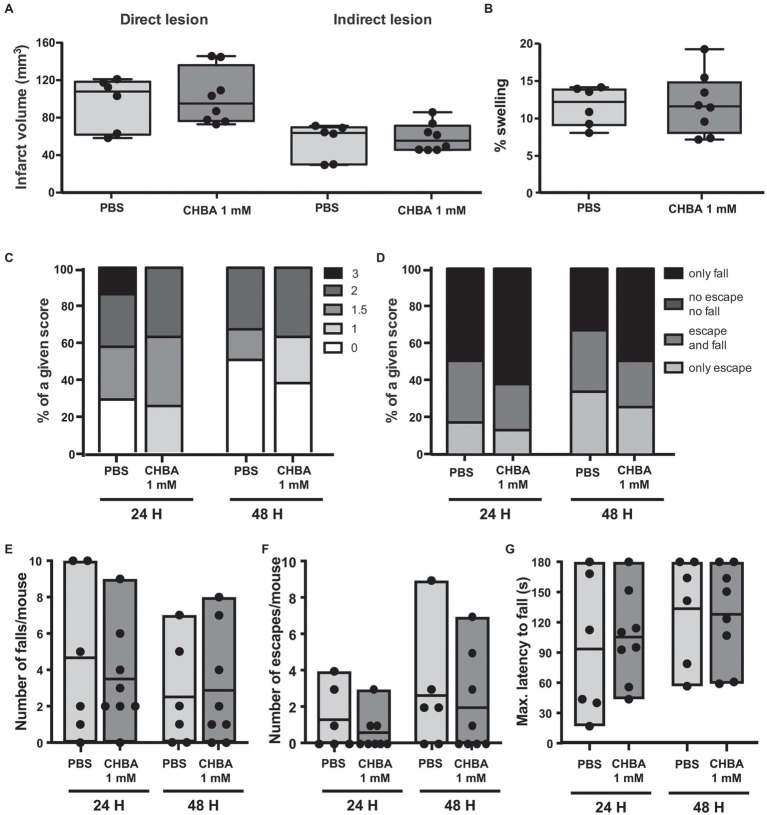
Effect of the intracerebroventricular (ICV) administration of the lactate receptor agonist CHBA after 30 min MCAO. **(A)** Direct (left) and indirect (right) lesion volumes and **(B)** swelling volumes from brains of mice that received a 2 μl ICV injection of vehicle (PBS, *n* = 6) or 1 mmol/L CHBA (*n* = 8) at reperfusion. Measurements were done on cresyl violet-stained serial coronal sections of frozen brains from mice sacrificed 48 h after MCAO induction. To evaluate the functional outcome following ICV treatment with PBS or CHBA, mice were scored for **(C)** neurological deficit (neuroscores: 0, no deficit; 1, failure to extend right forepaw; 1.5, intermittent circling; 2, circling; and 3, loss of circling or righting reflex), **(D)** overall performance on wire-hanging test, **(E)** number of falls from the wire, **(F)** number of times mice escaped from the wire, and **(G)** maximal latency time to fall from the wire. All the behavioral measurements were recorded at 24 h and 48 h after MCAO. Filled dots represent individual animals.

Several studies have shown *in vitro* effects of CHBA on cells of the central nervous system (namely, in neuronal and astrocytic cultures; Vardjan et al., 2018; de Castro Abrantes et al., 2019), but to date the only reported effects of CHBA *in vivo* come from experiments analyzing peripheral effects (Dvorak et al., 2012; Wallenius et al., 2017). We could not discard that the weak effects observed in our experiments might be related to the compound’s default in crossing the blood-brain barrier; hence, we decided to perform an experiment with ICV administration of the agonist. As the effective dose of CHBA *in vitro* is 100 times lower than the effective dose of L-lactate, and the drug is delivered directly to the brain, we chose to inject 1 mmol/L CHBA intracerebroventricularly, a concentration 100 times lower than that of L-lactate (Berthet et al., 2009; Figure 4). Similar to the experiments with IV administration, the ICV route did not show any significant effect on lesion size or neurological outcome.

**Figure 5:**
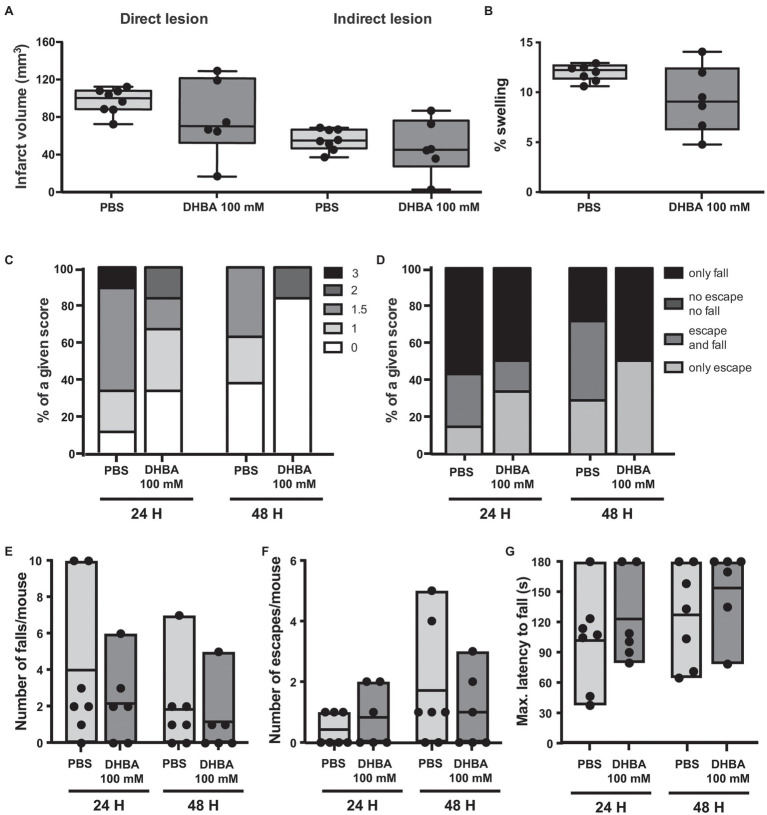
Effect of the ICV administration of the lactate receptor agonist 3,5-dihydroxybenzoic acid (DHBA) after 30 min MCAO. **(A)** Direct (left) and indirect (right) lesion volumes and **(B)** percentage of swelling from brains of mice that received a 2 μl ICV injection of vehicle (PBS, *n* = 8) or 100 mmol/L DHBA (*n* = 6) at reperfusion. Measurements were done on cresyl violet-stained serial coronal sections of frozen brains from mice sacrificed 48 h after MCAO induction. To evaluate the functional outcome following ICV treatment with PBS or DHBA, mice were scored for **(C)** neurological deficit (neuroscores: 0, no deficit; 1, failure to extend right forepaw; 1.5, intermittent circling; 2, circling; and 3, loss of circling or righting reflex), **(D)** overall performance on wire-hanging test, **(E)** number of falls from the wire, **(F)** number of times mice escaped from the wire, and **(G)** maximal latency time to fall from the wire. All the behavioral measurements were recorded at 24 h and 48 h after MCAO. Filled dots represent individual animals. Only seven control mice were taken into account for the wire-hanging test.

Another HCAR1 agonist, the less potent DHBA, which has an apparent EC_50_ of 0.15 mM (Liu et al., 2012), has been shown to exert effects similar to lactate in cultured neurons and acute hippocampal slices (Bozzo et al., 2013; Herrera-Lopez and Galvan, 2018). Previous work from our laboratory showed that DHBA elicited neuroprotection in hippocampal organotypic slices subjected to OGD when used at the same dose as L-lactate (Castillo et al., 2015). In light of our previous results with CHBA, and to ensure delivery to the brain, we directly tested DHBA in our MCAO model using ICV administration of the agonist at 100 mmol/L, the same concentration used for L-lactate (Figure 5). Again, no significant effects were observed on lesion size or neurological outcome. However, of note, the results of the DHBA treatment seem to point toward slightly better post-MCAO outcomes than those obtained using the more potent CHBA.

## Discussion

The administration of lactate receptor HCAR1 agonists to mice subjected to a transient ischemic insult did not appear to exert a protective effect in terms of lesion size or neurological outcome. This is in contrast to the effect of lactate administration (Berthet et al., 2009). At first, we considered the possibility that the lack of effect of CHBA, a potent HCAR1 agonist shown to activate the receptor *in vitro*, might have been related to its failure to reach the brain following intravenous administration. However, the absence of clear effects after the ICV administration of both CHBA and DHBA suggests that there might be other reasons.

The most straightforward explanation would be that lactate neuroprotection is mainly due to the nurturing effect offered by this metabolite. In opposition to the previous assumptions that associated elevated lactate with poor outcomes, we could rather think that increased production of lactate is an attempt of the brain to restore the calm after the storm. Then, the initial lactate paradox resolves, as it would not be surprising to observe a benefit when giving more of an endogenously produced good thing. Both endogenous and exogenous lactate could be used by astrocytes and suffering neurons to obtain anaplerotic precursors and to produce ATP (Jourdain et al., 2016; Magistretti and Allaman, 2018). At rest, under stimulation, and after intravenous lactate injection, lactate flows from astrocytes to neurons (Mächler et al., 2016; Zuend et al., 2020). And astrocytes with their end-feet in close contact with the blood vessels and lining the ventricles would most likely be the initial recipients of exogenously delivered lactate, *via* the monocarboxylate transporters (MCTs) MCT1 and MCT4. One of the sources of astrocytic-produced lactate is glycogen. Following astrocytic stimulation, lactate can be produced from internal glycogen stores, released to the extracellular space, and be further taken up by neurons *via* MCT2 to sustain their activity in response to the stimulus (Dinuzzo et al., 2012; Goncalves et al., 2018; Magistretti and Allaman, 2018). The addition of exogenous lactate could thus prevent the depletion of these stores while providing astrocytes with plenty of energy and building blocks to sustain survival and further proliferation. Moreover, this abundance of lactate could be shared with the neurons, allowing them to convert it into pyruvate, which would enter the TCA cycle and generate enough ATP to prevent neuronal swelling due to Na^+^/K^+^-ATPase failure (Magistretti and Allaman, 2018). This process also generates NADH, which has been proposed to influence NMDA receptor excitability (Jourdain et al., 2018). It could also allow the neurons to spare blood-borne glucose for energy production and drive it to feed the pentose phosphate pathway and provide NADPH that would improve their redox buffering capacity (Herrero-Mendez et al., 2009).

In support of the mainly metabolic effect of lactate, we have shown recently that IV administered lactate is metabolized very rapidly in the ischemic mouse brain. Using magnetic resonance spectroscopy after hyperpolarized [1-^13^C] lactate administration at therapeutic doses, we observed a peak of ^13^C-lactate followed within seconds by peaks of ^13^C-labeled pyruvate and bicarbonate in the brain (Hyacinthe et al., 2020), implying intracellular transport and metabolism of the exogenous lactate. Consistently, and even following intraperitoneal injection, when administered after induction of hypoxia-ischemia in rat pups, [3-^13^C]-labeled lactate rapidly reached the brain, with label incorporation into several metabolites reflecting its use on the TCA (Roumes et al., 2020). Further stressing the importance of the metabolic effect of lactate, in the same neonatal hypoxia-ischemia model, the inhibition of lactate dehydrogenase, the enzyme required to convert lactate into pyruvate, prevented lactate neuroprotection, and there was decreased oxidative damage and a reduction of the cytotoxic edema when administered after the insult (Roumes et al., 2020). Remarkably, the infusion of hypertonic lactate to traumatic brain injury patients increased the brain levels of pyruvate and glucose while leading to a better outcome (Bouzat et al., 2014; Carteron et al., 2018).

D-lactate is a partial agonist of HCAR1 (Cai et al., 2008) that can be metabolized in the brain similarly to L-lactate (Adeva-Andany et al., 2014; Castillo et al., 2015). Similarly to L-lactate, D-lactate has also been shown to protect from the deleterious effects of hypoxia both *in vivo* and *in vitro* (Castillo et al., 2015). However, neurons cannot directly transport D-lactate due to its weak affinity for MCT2, the main neuronal transporter (Halestrap, 2012). Conversely, endothelial cells and astrocytes, both expressing MCT1, could import and likely metabolize D-lactate. The partial HCAR1 agonist D-lactate, applied at doses similar to those used for neuroprotection was not able to induce changes in the firing frequency of neurons (Herrera-Lopez and Galvan, 2018), although it decreased the neuronal calcium transient frequency (Bozzo et al., 2013). Intriguingly, pyruvate, which is also transported by MCTs and metabolized similarly to lactate, but does not activate HCAR1, induces significant protection against OGD in hippocampal slice cultures but not in the MCAO model (Castillo et al., 2015) and does not protect in the neonatal HI model (Roumes et al., 2020).

Based on the data from *in vitro* experiments, one would expect a protective effect following administration of the lactate receptor agonists, as the extracellular activation of neuronal HCAR1 would slow down neuronal activity (Bozzo et al., 2013; Herrera-Lopez and Galvan, 2018; de Castro Abrantes et al., 2019), exacerbated after the ischemia. Whereas at present we do not have data on the activity of neurons after *in vivo* administration of the agonists, after analyzing the lesion volumes and the behavioral output, we observed that the agonists failed to show any measurable protection from the ischemic damage. Some reports have proposed the existence of other putative lactate receptors, with Gs activity instead of the HCAR1 Gi activity (Tang et al., 2014; Vardjan et al., 2018; D’Adamo et al., 2021). However, the evidence and characterization of these receptors still remain elusive (Mosienko et al., 2018). Others have proposed the interaction of the HCAR1 Giβγ moiety with other GPCRs, and the activation of ERK1/2, PI3K, and Akt pathways following HCAR1 stimulation (Li et al., 2014; de Castro Abrantes et al., 2019; Herrera-López et al., 2020). Additionally, it is not to be excluded that the HCAR1 agonists might interact with receptors other than HCAR1, which might hamper the protection foreseen in relation to HCAR1 activation.

An additional explanation for the lack of protective effect of the agonists in our model, which may help reconcile the evidence provided by *in vivo* and *in vitro* data, could be the interference of the agonists with endogenous lactate. It is well known that the endogenous brain lactate concentration following hypoxia-ischemia shows a fast increase that remains elevated for a long time (Harada et al., 1992; Lei et al., 2009; Alf et al., 2012; Hyacinthe et al., 2020). However, not only hypoxia increases brain lactate concentration. Our experiments were done using isoflurane as an anesthetic. The exposure of mice to isoflurane causes a significant increase in brain lactate concentration (Horn and Klein, 2010; Zuend et al., 2020). Lactate has even been shown to increase in the medium of cells treated with isoflurane (Brabec et al., 1984). The isoflurane-released lactate could be related to the protective effect that it exerts from ischemic insults (Berthet et al., 2009; Burchell et al., 2013; Horn and Klein, 2013), and we had observed that exogenously administrated lactate had a stronger protective effect when using ketamine/xylazine anesthesia compared to isoflurane (Berthet et al., 2009). The presence of isoflurane is an evident difference between our *in vivo* and *in vitro* experiments, which could affect HCAR1 responsiveness to the agonists. Moreover, the isoflurane-driven increase in lactate could lead to relevant changes that could affect lactate transport even before the administration of the drugs. Indeed, animals undergoing sham surgery under isoflurane anesthesia have been shown to experience changes in MCTs, with the increased expression on vessels (Rosafio et al., 2016), suggesting that maybe exposure to isoflurane and the subsequent changes in brain lactate concentrations are sufficient to trigger changes in the distribution of the lactate transporters. The increase in the intracerebral lactate concentration due to the anesthetic, adding up to the lactate surge due to the hypoxia-ischemia could set the stage for an already elevated concentration of lactate. While additional lactate administration still may provide protection, the administration of the more potent HCAR1 agonists added to an already high lactate concentration could bring a different scenario.

The agonists, which are not transported into cells and could thus remain in the interstitial space before being cleared out, might reach high local concentrations near HCAR1 receptors. Although there are no pharmacokinetic data available for the two compounds tested in these experiments, a nutritional study measuring urinary excretion of DHBA after cereal ingestion demonstrated a peak at 10 h, suggesting a long bioavailability (Zhu et al., 2014). At rest, there is a basal tone of HCAR1, with a mild inhibition of adenylate cyclase (AC). Due to the ischemic insult, the steady increase in lactate concentration can reach a level where the receptor is fully activated (Cai et al., 2008; Liu et al., 2009), inhibiting AC and decreasing cAMP levels. The short sustained stimulation of GPCRs like HCAR1 can result in a decreased response over a time frame of minutes due to transducer uncoupling or acute desensitization (Rajagopal and Shenoy, 2018). Further, if the stimulation is sustained over hours, this may result in decreased receptor expression at the plasma membrane due to downregulation or prolonged desensitization (Kelly et al., 2008; Rajagopal and Shenoy, 2018). Indeed, HCAR1 has been shown to internalize after prolonged exposure to agonists *in vitro* (Liu et al., 2009). Therefore, if localized extracellular levels of the agonists (endogenous and/or exogenous) are large enough, a diminished response of HCAR1 could be expected. Supporting this idea, it has been reported that lactate or agonist stimulation of neuronal HCAR1 has opposite effects on the firing frequency depending on their concentrations, with large concentrations giving rise to increased excitability (Herrera-Lopez and Galvan, 2018). Moreover, very high doses of lactate applied after OGD *in vitro* lose the protective effect and can be even toxic (Berthet et al., 2009).

In our MCAO-operated mice, the cumulative presence of a substantial amount of endogenous lactate and potent HCAR1 agonists could have tipped the receptor activation toward hyperexcitability. The overstimulation of the neuronal HCAR1 in the acute post-stroke phase could therefore, lead to a detrimental exacerbation of neuronal activity, instead of slowing it down to balance the effects caused by the excessive extracellular glutamate that accumulates after hypoxia-ischemia. In earlier unpublished experiments, we failed to improve the neurological outcome in mice with short-term repeated lactate intravenous administration, while single administration was efficient (Berthet et al., 2009; Castillo et al., 2015; Buscemi et al., 2020), suggesting that protracted receptor stimulation was not beneficial. As mentioned before, the protective effects of exogenously administered lactate were stronger when using ketamine/xylazine anesthesia compared to isoflurane. The neuroprotective effects observed when using the agonist DHBA after OGD, an *in vitro* system (Castillo et al., 2015), could be related to the medium change allowing recovery after the OGD, which might have cleared out enough hypoxia-produced lactate to eliminate interference with the agonist and thereby not prevent receptor-mediated protective effects. Although the discrepancy between the effects of DHBA on the *in vitro* vs. *in vivo* models could also be related to the differences between the cellular architecture of organotypic cultures and that of the whole organism, particularly on vascular-related aspects like the presence/absence of circulation, endovascular compartment and blood-brain-barrier. Still, in our *in vivo* experiments, whereas the more potent agonist CHBA tends toward a more deleterious effect, the observed effects after the treatment with the weaker DHBA hint at a slight amelioration of the behavioral outcome.

The present results with the HCAR1 agonists cannot exclude that the beneficial effects of exogenous lactate administration after MCAO could be majorly attributable to a metabolic effect, nor absolutely discard any neuroprotective effect of HCAR1 stimulation. It would be interesting thus to test the effects of exogenous lactate administration to HCAR1 KO mice subjected to MCAO, to use a different anesthetic and/or a delayed administration of the agonists to wild-type mice. Finally, MCT1, MCT2, and MCT4 (Rosafio et al., 2016) lactate transporters show changes in expression and cellular localization after MCAO, which could contribute to lactate’s protective mechanism and may be worth a future study.

## Data Availability Statement

The raw data supporting the conclusions of this article will be made available by the authors, without undue reservation.

## Author Contributions

LB, PM and LH contributed to the study conception. LB and LH drafted the manuscript and figures. LB and CB acquired and analyzed the data. All authors contributed to the article and approved the submitted version.

### Conflict of Interest

The authors declare that the research was conducted in the absence of any commercial or financial relationships that could be construed as a potential conflict of interest.
